# Nanotwins Strengthening High Thermoelectric Performance Bismuth Antimony Telluride Alloys

**DOI:** 10.1002/advs.202200432

**Published:** 2022-03-18

**Authors:** Haixu Qin, Wanbo Qu, Yang Zhang, Yongsheng Zhang, Zihang Liu, Qian Zhang, Haijun Wu, Wei Cai, Jiehe Sui

**Affiliations:** ^1^ State Key Laboratory of Advanced Welding and Joining Harbin Institute of Technology Harbin 150001 China; ^2^ State Key Laboratory for Mechanical Behavior of Materials Xi'an Jiaotong University Xi'an 710049 China; ^3^ Instrumental Analysis Center Xi'an Jiaotong University Xi'an 710049 China; ^4^ Key Laboratory of Materials Physics Institute of Solid State Physics Chinese Academy of Sciences Hefei 230031 China; ^5^ Department of Materials Science and Engineering Harbin Institute of Technology (Shenzhen) Shenzhen 518055 China

**Keywords:** Bi_2_Te_3_, mechanical properties, nanotwins, thermoelectric performance

## Abstract

Bi_2_Te_3_ based thermoelectric alloys have been commercialized in solid‐state refrigeration, but the poor mechanical properties restrict their further application. Nanotwins have been theoretically proven to effectively strengthen these alloys and could be sometimes constructed by strong deformation during synthesis. However, the obscure underlying formation mechanism restricts the feasibility of twin boundary engineering on Bi_2_Te_3_ based materials. Herein, thorough microstructure characterizations are employed on a series of Bi_0.4_Sb_1.6_Te_3+_ *_*δ*_* alloys to systematically investigate the twins’ formation mechanism. The results show that the twins belong to the annealing type formed in the sintering process, which is sensitive to Te deficiency, rather than the deformation one. The Te deficiency combined with mechanical deformation is prerequisite for constructing dense nanotwins. By reducing the *δ* below −0.01 and undergoing strong deformation, samples with a high density of nanotwins are obtained and exhibit an ultrahigh compressive strength over 250 MPa, nearly twice as strong as the previous record reported in hierarchical nanostructured (Bi, Sb)_2_Te_3_ alloy. Moreover, benefitting from the suppressed intrinsic excitation, the average *zT* value of this robust material could reach near 1.1 within 30–250 °C. This work opens a new pathway to design high‐performance and mechanically stable Bi_2_Te_3_ based alloys for miniature device development.

## Graphical abstract

This study reports a great advance in strengthening (Bi, Sb)_2_Te_3_ with high thermoelectric performance solely through nanotwins, an ultra‐high compressive strength over 250 MPa, nearly twice that of the previous record reported in hierarchical nanostructured (Bi, Sb)_2_Te_3_ alloy. It starts from clarifying the formation mechanism of twins and then manipulating high‐density nanotwins to synergistically optimize thermoelectric and mechanical properties.

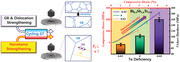

## 1 Introduction

Mechanical property governs the application prospect of both structural materials and functional materials in practice. In addition to the composition modification, the introduction of microstructural defects, such as dislocation and grain boundary, offers an alternative route to improve the overall mechanical properties based on the material's paradigm of “microstructure determines properties.”^[^ ^1^ ^]^ Grain refinement results in the hardening in alloys and ceramics that follows the empirical Hall‐Petch relation,^[^ ^2^, ^3^ ^]^ but the grain coarsening occurs at elevated temperature for nanostructured bulk materials due to the large driving force of high energy of high‐angle grain boundaries (GB).^[^ ^4^ ^]^ Meanwhile, since the high‐energy GBs are thermally unstable, their sliding or migration dominates the deformation mechanism in the heating process, leading to the reduced strength.^[^ ^5^, ^6^ ^]^ Constructing nanotwins is also an effective method to strengthen the materials,^[^ ^7^ ^]^ which has been experimentally verified in metals, diamonds, and boron nitrides.^[^ ^8^, ^9^, ^10^ ^]^ The excess energy of coherent twin boundary is typically about one order of magnitude lower than that of grain boundaries,^[^ ^11^ ^]^ which makes nanotwinned structures energetically more stable than the nanograined counterparts.

Thermoelectric (TE) materials, which directly realize the conversion between heat and electricity without any emissions or moving parts, have attracted increasing attention in the field of both power generation and solid‐state refrigeration.^[^ ^12^, ^13^, ^14^ ^]^ The performance of the TE devices is mainly determined by the dimensionless figure of merit, *zT* = *S* ^2^ *σT*/*κ*, where *S* is the Seebeck coefficient, *σ* the electrical conductivity, *T* the absolute temperature, and *κ* the thermal conductivity mainly consisting of electronic and lattice parts (*κ* = *κ*_e_ + *κ*_l_).^[^ ^15^, ^16^ ^]^ Bi_2_Te_3_ based alloys, classical commercial TE materials, have been widely used for TE refrigeration or power generation in the temperature range of 30–200 °C. Traditionally, alloying with isostructural Sb_2_Te_3_ or Bi_2_Se_3_, the *zT* value of both the p‐type and n‐type Bi_2_Te_3_ based alloys prepared by unidirectional solidification techniques could reach around 1.0.^[^ ^17^, ^18^ ^]^ And more recently, their TE performance has been further improved via microstructure engineering which significantly reduces the lattice thermal conductivity by hindering the heat transfer.^[^ ^19^, ^20^, ^21^ ^]^ Typically, Kim et al. proposed the method of liquid‐phase compaction to improve the *zT* value of p‐type Bi_0.5_Sb_1.5_Te_3_ alloy up to an unprecedented value of 1.85.^[^ ^22^ ^]^ Then, Wu et al. and Zhu et al. also prepared n‐type Bi_2_Te_3_‐Bi_2_Se_3_ solutions by a similar method and obtained a high *zT* value of ≈1.4.^[^ ^18^, ^23^ ^]^ The significantly enhanced TE properties will facilitate the wider applications of Bi_2_Te_3_ based alloys in the solid‐state refrigeration field.

**Figure 1:**
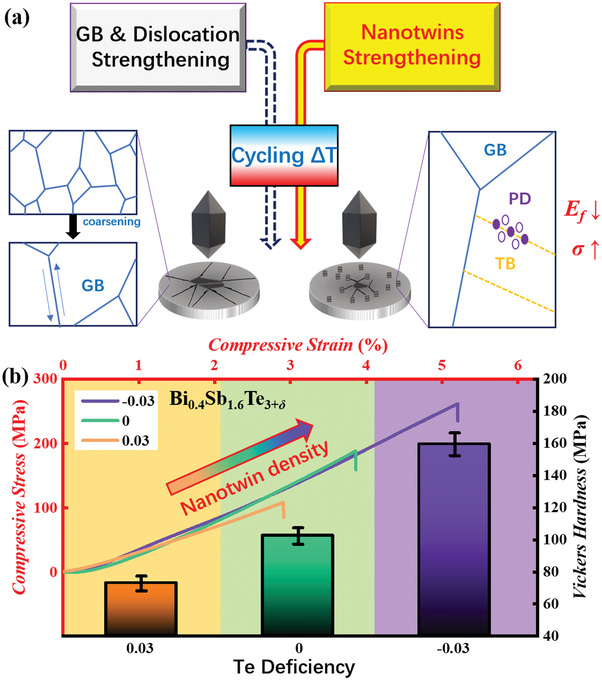
The nanotwin‐induced strengthening strategy. a) the schematic diagram of the underlying mechanism of nanotwin‐induced strengthening in layered‐structured Bi_2_Te_3_, compared with traditional grain boundary strengthening. b) Strengthening via the increase of nanotwin density through raising Te deficiency.

In practical engineering conditions, TE materials often suffer from unavoidable thermomechanical stresses from the cycling of the temperature gradients.^[^ ^24^, ^25^, ^26^ ^]^ Hence, mechanical strength is of equal importance to TE performance for guaranteeing service stability. Furthermore, the miniature Bi_2_Te_3_ based thermoelectric devices have attracted more attention due to their potentials for being applied in electronic, optoelectronic, and bioanalytical devices,^[^ ^27^, ^28^, ^29^, ^30^ ^]^ such as microprocessors, semiconductor lasers, and DNA micro‐arrays, which puts forward much severer standards for the mechanical strength to machine the Bi_2_Te_3_ based materials into micron‐scale or even smaller size. However, the crystal structure of Bi_2_Te_3_ based alloys composes of periodically stacked quintuple atomic layers (Te1‐Bi‐Te2‐Bi‐Te1) along the *c* axis, and the adjacent atomic layers are bound with van der Waals force (**Figure** **1**).^[^ ^31^ ^]^ Such weak physical bonding makes the materials easily suffer a cleavage fracture and therefore possesses native poor mechanical strength,^[^ ^32^, ^33^ ^]^ leading to problems of difficult machining and unstable serving. Grain refinement by mechanical alloying^[^ ^21^ ^]^ or melt spinning method^[^ ^34^ ^]^ offers a general pathway to suppressing the cleavage problem commonly found in traditional zone melt ingots and enhancing the mechanical strength, but as we previously discussed, the thermodynamically unstable characteristic of high‐energy (GBs) limits the Hall‐Petch effect. Moreover, twin boundaries allow more advantageous electron migration characteristics than ordinary grain boundaries while effectively hinder phonon transport.^[^ ^35^ ^]^ In the past decade, nanotwins have been reported in these Bi_2_Te_3_ based alloys prepared by ball milling followed by hot pressing.^[^ ^36^, ^37^, ^38^, ^39^, ^40^ ^]^ Besides, Hayashi et al. also reported the occurrence of twins in the Bi_1.9_Sb_0.1_Te_2.7_Se_0.3_ prepared by equal channel angular extrusion.^[^ ^41^ ^]^ In these works, the twins are thought to be generated from the severe deformation, similar to the deforming twins observed in face‐centered cubic (FCC) crystals with nanocrystal grains.^[^ ^42^, ^43^ ^]^ However, not all the samples experiencing severe deformation process exhibits dense nanotwins,^[^ ^19^, ^21^, ^44^ ^]^ indicating that other factors also influence the twins’ formation process.

For the Bi_2_Te_3_ based alloys, since the Te is very unstable and easy to volatilize or precipitate out as second phases in the fabrication process,^[^ ^45^, ^46^, ^47^ ^]^ it is very difficult to guarantee the samples prepared by the same method in different groups to possess the same Te content. Hence, the composition, i.e., Te content, might be another factor that affects the twins’ formation. Herein, through the traditional melting method followed by ball milling and spark plasma sintering, we prepare a series of p‐type Bi_0.4_Sb_1.6_Te_3+_ *_*δ*_* (−0.03 < *δ* < 0.03) alloys to systematically investigate the formation mechanism of nanotwins, confirming the twins are formed in the sintering process and discovering both the severe deformation and Te‐poor condition account for the dense twins’ construction. As a result, nanotwins are universally observed in these samples with *δ* < 0 and effectively improve compressive strength from 189 MPa for Bi_0.4_Sb_1.6_Te_3_ to over 250 MPa for Bi_0.4_Sb_1.6_Te_2.98/2.97_, as revealed by the schematic diagram (Figure 1). Better still, decreasing the Te content is in favor of the antisite defects (Bi(Sb)_Te_) formation, which increases the carrier concentration and suppresses the bipolar effect to producing a high average *zT* value above 1.0 within 30–250 °C, making this mechanically robust material very promising for the practical application.

## 2 Results and Discussions

**Figure 2:**
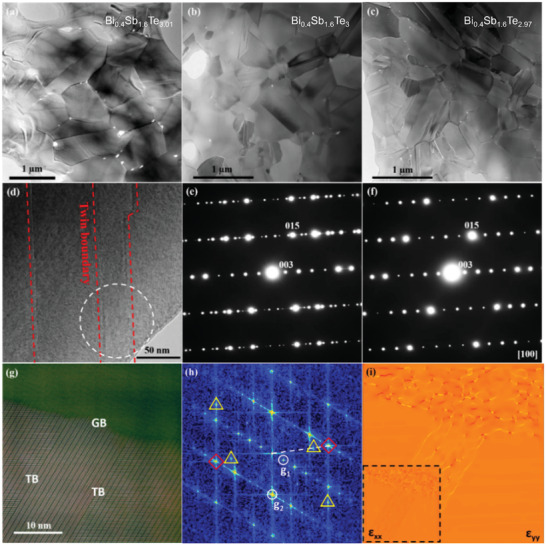
Low‐magnification TEM image of a) Bi_0.4_Sb_1.6_Te_3.01_, b) Bi_0.4_Sb_1.6_Te_3_, and c) Bi_0.4_Sb_1.6_Te_2.97_ showing sub‐micrometer scaled grains with a high density of twins. d) HRTEM image showing four twins with three twin boundaries. e) Electron diffraction pattern from the twin boundary of (d) showing a typical reflection of twins. f) Electron diffraction pattern from twin interior, for reference. g) Atomically resolved STEM HAADF image focusing on the curved grain boundaries and straight twin boundaries, which terminated at the grain boundaries. h) FFT image of (g) showing the crystallographic orientation. i) Strain map profiles *ε*_xx_ using the g1 and g2 reflections of one twin variant marked in (h), the inset is the strain map profiles *ε*_yy_.

The as‐sintered Bi_0.4_Sb_1.6_Te_3+_ *_*δ*_* samples with 95–98% relative to theoretical density are crystallized in rhombohedral structure evidenced by X‐ray diffraction (XRD) patterns shown in Figure S1 (Supporting Information). All the diffraction peaks match well with the standard pattern of *JCPDS* 72–1836 with no secondary phase detected, confirming that modulating the Te content within appropriate limits does not change the crystal structure. The microstructures of Bi_0.4_Sb_1.6_Te_3.01_, Bi_0.4_Sb_1.6_Te_3,_ and Bi_0.4_Sb_1.6_Te_2.97_ are further studied by (scanning) transmission electron microscope (TEM/STEM). As shown in low‐magnification TEM images (**Figure** **2**a–c), the grain size of the three samples are in a similar range, from hundreds of nanometers to several micrometers. Their key difference is the density of nanotwins, i.e., a high density in Bi_0.4_Sb_1.6_Te_2.97_ while a lower density in Bi_0.4_Sb_1.6_Te_3_ and very scarce in Bi_0.4_Sb_1.6_Te_3.01_. The high‐resolution TEM image in Figure 2d presents four nanotwins with three twin boundaries. The electron diffraction patterns from the twin boundary, shown in Figure 2e, present the crystallographic relation of the twins, i.e., the twin plane is along (003), compared with the diffraction patterns from the twin interior in Figure 2f. The atomically resolved high angle annular dark field (STEM HAADF) image in Figure 2g presents a region possessing curved grain boundaries and two straight twin boundaries, the latter of which terminated at the grain boundaries. It is clearly shown that the twin boundaries are coherent while the grain boundaries are always semi‐coherent. Figure 2h shows the fast Fourier transform (FFT) image of Figure 2g, which reveals the two grains possess a sharing plane, as marked with the yellow dashed line. To reveal the strain state of the grain boundary and twin boundaries, the high‐quality HR‐HAADF image was analyzed by geometric phase analysis (GPA),^[^ ^48^ ^]^ which is a semi‐quantitative lattice image‐processing approach for revealing spatial distribution of relative elastic strain. The strain analysis result shows that there is little strain variation between twin variants, while large strain variation appears between grains. Both the grain boundaries and twin boundaries are effective phonon scattering centers, while twin boundaries could maintain the carrier transport better than grain boundaries.

**Figure 3:**
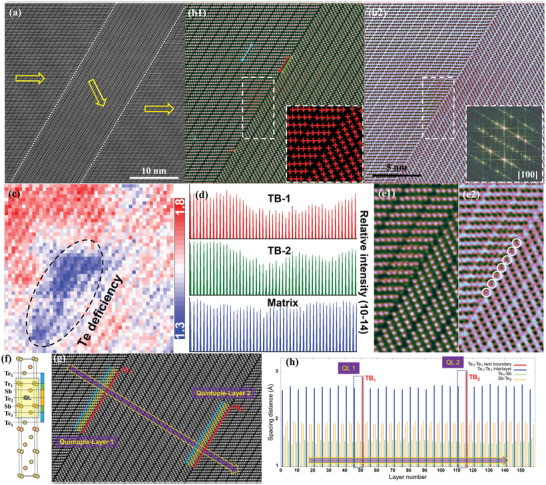
a) Atomically resolved STEM HAADF image showing two twin boundaries. b1,b2) Atomically resolved STEM HAADF and ABF image focusing on one twin boundary. The inset in (b1) showing peaks quantitatively found through Gaussian Fitting; the inset in (b2) is the FFT image of (b1,b2). c) Intensity mapping of (b1) showing a cluster of Te deficiency around the twin boundary. d) Intensity line scans from marked atom arrows marked in (b1), showing the decreased intensity of Te deficient twin boundary compared with the matrix. e1,e2) Enlarged image from the twin boundary as marked in (b1,b2), showing Bi/Sb interstitials at the twin boundary, close to the Te‐deficient atom columns. f) Structural model of Sb_2_Te_3_ showing the quintuple‐layers (QL) with ‐Te1‐Sb‐Te2‐Sb‐Te1‐ sequence. g) Atomically resolved STEM HAADF image showing two twin boundaries. h) Layer spacing profile along the arrow marked in (g). Colors demarcating different kinds of layer spacings were corresponding to (f)–(h).

To reveal the detailed structures around twin boundaries in Bi_0.4_Sb_1.6_Te_2.97_, we employed qualitative and even quantitative analysis based on aberration‐corrected STEM. Most regions of twin boundaries are perfectively coherent as shown in **Figure** **3**a, while some regions present Te‐deficiency nanoclusters and interstitials at the boundaries, as analyzed below. Figure 3b1,b2 shows simultaneously acquired STEM HAADF and ABF (annular bright field) images, focusing on one twin boundary. This boundary possesses one nanocluster, which can be seen more clearly in the STEM ABF imaging mode due to the strong strain effect. To analyze the nanosheets, we first identify the atom positions and their intensities quantitatively through Gaussian fitting,^[^ ^49^, ^50^, ^51^ ^]^ as shown in the inset of Figure 3b. Figure 3c is the intensity mapping of Figure 3b1, showing a cluster of Te deficiency around the twin boundary. The intensity profiles shown in Figure 3d, obtained from line scans of marked Te1 atom column, present the decreased intensity of Te around the twin boundaries compared with the twin interior (matrix). The Te‐deficient twin boundary is the characteristic of materials with lower Te content. In addition, adjacent to the Te deficient cluster, interstitials can also be observed at the twin boundary,^[^ ^52^ ^]^ as shown in Figure 3e1,e2, accounting for enhanced phonon scattering together with the Te vacancies.^[^ ^53^ ^]^ Considering that decreasing the Te content generates excess Bi or Sb, these interstitials might be these two atoms. Although Bi or Sb generally cannot enter into the gap between quintuple‐layers due to their large radius, the larger layer spacings discussed below in twin boundaries make it possible. Figure 3f is the structural model of R‐3m Sb_2_Te_3_ and it shows that there are three types of layer spacings among the quintuple‐layers (QLs), Te1‐Sb, Sb‐Te2 in a QL, and Te1‐Te1 between QLs.^[^ ^54^ ^]^ In addition, at the twin boundary, there is another lattice spacing, the so‐called Te1‐Te1 at the twin boundary. To evaluate the microscopic law of layer spacing and the potential relationship between twin boundaries and interstitials, we quantitatively calculated them based on the atom positions of Figure 3g. Figure 3h is the layer spacing profile along the arrow marked in Figure 3g, i.e., the c direction. It is clearly shown that the layer spacings also follow a quintuple periodical arrangement along c direction like atomic layers, and the layer spacings of the above four conditions are 0.15, 0.19, 0.26, and 0.29 nm for Te1‐Sb, Sb‐Te2, Te1‐Te1 between QLs and Te1‐Te1 at the twin boundary. The layer spacing at the twin boundary is wider (≈10%) than that between common QLs, which makes it possible for the formation of Bi or Sb interstitials.

As shown in the above TEM images, all the nanotwins were intersected through the whole grains, which is a characteristic of annealing twins, different from the nonintersected deformation twins. Hence, the nanotwins should be generated in the sintering process, rather than in the ball milling process reported in previous studies.^[^ ^36^ ^]^ To figure out in which process the nanotwins are formed, ball‐milled Bi_0.4_Sb_1.6_Te_2.97_ powders are sintered under a low temperature of 473 K followed by the microstructure characterization. The grains of this sample exhibit a very small size (lower than 50 nm) due to the low sintering temperature, and no trace of twins can be detected (Figure S2a, Supporting Information). Some nanotwins can be observed in these grains with much larger size (> 100 nm) (Figure S2b, Supporting Information), indicating the twins are formed in the grain‐growing process, i.e., the twins observed in Figure 1 indeed belong to annealing twins which are generated in the sintering process. Nevertheless, the ball milling is also necessary for the twins’ formation, the intense stress induced by the heavy deformation could provide the driving force for recrystallization. Twins cannot be constructed in the sample prepared by traditional melting followed by hand milling and SPS process (Figure S3, Supporting Information).

Then, density functional theory (DFT) calculations are conducted to simulate the formation of stacking faults in Bi_0.4_Sb_16_Te_3_. The stacking faults energy *E*_f_ is vital for twins’ formation, where low *E*_f_ means more favorable for the occurrence of twins. Since the sample always keeps in Te‐poor condition even in these samples with *δ* > 0 (reported in our previous work^[^ ^55^ ^]^), there are only two cases that should be considered (Figure S4, Supporting Information), 1) stoichiometric Bi_0.4_Sb_1.6_Te_3_ and 2) Bi_0.4_Sb_1.6_Te_3_ with low Te content. For case two (2), it should be noted that Te vacancy is unstable due to the high formation energy and inclined to be occupied by the Bi or Sb atoms.^[^ ^56^ ^]^ Hence, three compositions composed of Bi_0.4_Sb_1.6_Te_3_, (Bi_0.4_Sb_1.6_)(Bi_0.3_Te_2.7_), and (Bi_0.4_Sb_1.6_)(Sb_0.3_Te_2.7_) are employed in the simulation process, obtaining the stacking fault energy of 48.9, 57.7, and 38.5 mJ m^−2^, respectively. The detailed calculation process and results are present in Support Information III.

According to the simulation results, the intrinsic *E*_f_ of stoichiometric Bi_0.4_Sb_1.6_Te_3_ is low enough to support the twins’ formation. Although reducing the Te content and occupying the Te vacancies by some Sb atoms slightly reduces the *E*_f_, there should not be a large difference in the twin density between Bi_0.4_Sb_1.6_Te_3.01_ and Bi_0.4_Sb_1.6_Te_2.97_. That means the twin density distinction in the samples with different Te content is not induced by the *E*_f_ variation. For the annealing twins’ formation, the growth accident model,^[^ ^57^, ^58^, ^59^ ^]^ which asserts that a coherent twin boundary forms at a migrating grain boundary because of stacking faults, is supported by a majority of recent experimental results, indicating that the stacking faults is the prerequisite for the twins’ formation. However, the accompanying strain and boundaries energy always lead to an energy increase for the system with the occurrence of stacking faults. Hence, the stacking faults are thermodynamic nonequilibrium defects and are inclined to disappear when the displaced atoms have enough energy to overcome the kinetic barrier. It has been reported that the Te in Bi_2_Te_3_‐Sb_2_Te_3_ based binary compounds even grown from stoichiometric melts tends to precipitate as a secondary phase.^[^ ^45^, ^46^ ^]^ These second phases with low melting points can melt in the followed sintering process and enhance the atoms’ migration ability near the grain boundary,^[^ ^22^ ^]^ which could help the displaced atoms in stacking faults return to their normal position and impede the twins’ formation. In addition, the melted Te‐rich phase also leads the grain to grow much larger,^[^ ^23^, ^60^ ^]^ during which large grains consume small grains and their twins without producing new twin boundaries.^[^ ^58^ ^]^ For the Bi_0.4_Sb_1.6_Te_3+_ *_*δ*_* alloys with *δ* < 0, the Te‐rich phase is accordingly eliminated by reducing the Te content, which clears away the obstruction for twins’ formation and generates dense nanotwins. However, for these samples with *δ* > 0, more Te‐rich phases occur, thus leading to the rare presence of nanotwins. In conclusion, the twins’ formation premise both the heavy deformation and the absence of Te‐rich phases.

**Figure 4:**
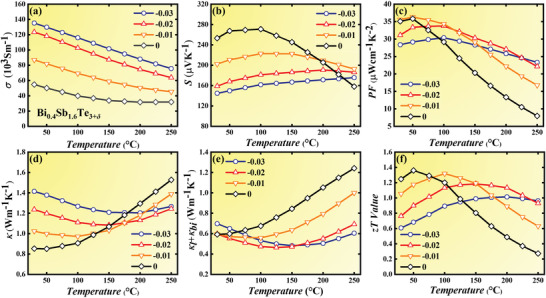
Temperature‐dependent thermoelectric properties of as‐sintered Bi_0.4_Sb_1.6_Te_3+_ *_*δ*_*. a) Electrical conductivity, b) Seebeck coefficient, c) power factor, d) thermal conductivity, e) sum of lattice and bipolar thermal conductivity, and f) *zT* value.

Adding some excess Te (*δ* > 0) could modify the carrier transport characteristics and reduce the lattice thermal conductivity to produce an ultra‐high *zT* value, which has been reported in our previous work.^[^ ^55^ ^]^ For the sample with Te content of *δ* < 0, the electrical conductivity *σ* in **Figure** **4**a displays typical semi‐metal features, i.e., decreases with the temperature. Then, by reducing the Te content, sharp *σ* enhancement is achieved, from 54.7 × 10^3^ S m^−1^ for Bi_0.4_Sb_1.6_Te_3_ to 137.5 × 10^3^ S m^−1^ for Bi_0.4_Sb_1.6_Te_2.97_, which mainly results from that decreasing the Te content produces more antisite defects Bi(Sb)_Te_ to generate a large number of holes, verified by the Hall carrier concentration measurement listed in Table S1 (Supporting Information). However, the twin boundaries might impede the carrier transport. According to data reported in our previous work,^[^ ^55^ ^]^ the Bi_0.4_Sb_1.6_Te_3.01_ exhibits a similar carrier concentration with the Bi_0.4_Sb_1.6_Te_2.99_ (2.37 × 10^19^cm^−3^ for Bi_0.4_Sb_1.6_Te_3.01_ and 2.48 × 10^19^cm^−3^ for Bi_0.4_Sb_1.6_Te_2.99_), while the carrier mobility of Bi_0.4_Sb_1.6_Te_3.01_ are 36% higher than that of Bi_0.4_Sb_1.6_Te_2.99_ (303.1 cm^2^V^−1^s^−1^ for the Bi_0.4_Sb_1.6_Te_3.01_ and 222.0 cm^2^V^−1^s^−1^ for the Bi_0.4_Sb_1.6_Te_2.99_). The only difference between these two samples is that the Bi_0.4_Sb_1.6_Te_2.99_ presents much higher twin density than the Bi_0.4_Sb_1.6_Te_3.01_, shown in Figure 2 a,b. Hence, we deduce that the twin boundaries might enhance the electron‐scattering and thus leading to reduced carrier mobility. Figure 4b presents the Seebeck coefficient *S* with a positive value, indicating the holes dominate the electrical transport properties. And due to the negative correlation between *S* and *n*, the samples with lower Te content possess much smaller *S* values than the pristine sample. Nevertheless, the larger *n* suppresses the bipolar effect and thus shifts the point corresponding to peak *S* value up to higher temperatures. Moreover, the increased carrier concentration could also induce the convergence of multivalley bands and increase the effective mass. The details are presented in Support Information IV. Ultimately, although the deteriorated *S* caused by heavy acceptor doping pulls down the room‐temperature power factor *PF*, the suppressed bipolar effect contributes to a *PF* enhancement as temperature increases, shown in Figure 4c. For instance, the *PF* of Bi_0.4_Sb_1.6_Te_2.97_ reaches 23.3 mW cm^−1^ K^−2^ at 250 °C, 195% higher than that of Bi_0.4_Sb_1.6_Te_3_.

The thermal conductivity *κ* also varies with the Te content. The significantly improved electrical conductivity with decreasing Te content brings about high electronic thermal conductivity (calculated by the *κ*_e_ = *LσT*), thus leading to obvious *κ* enhancement below 125 °C, shown as Figure 4d. However, things change as the temperature rises, a decline of *κ* occurs above 125 °C in these samples with lower Te content. To clarify it, the sum of lattice and bipolar thermal conductivity is obtained by subtracting the *κ*_e_ from the *κ* and shown in Figure 4e. The *κ*_b_ + *κ*_l_ of all samples first decreases with the temperature, and then quickly increases after the onset of the bipolar effect. Near room temperature, the *κ*_b_ can be ignored, thus *κ*_b_ + *κ*_l_ can be assumed as sole *κ*_l_ exhibiting an upward tendency with *δ* decreasing. Dense twin boundaries play important roles to enhance phonon‐scattering and should reduce the *κ*_l_, which has been verified in Zu et al.’s work.^[^ ^35^ ^]^ However, reducing the Te content provides more chance for the formation of antisite defects Bi(Sb)_Te_, which can be verified by the improved hole concentration listed in Table S1 (Supporting Information). Based on Hashibon et al.’s work about the native defects in Bi_2_Te_3_,^[^ ^56^ ^]^ the antisite defects are more inclined to be formed at Te1 sites. Since the electronegativity difference between Bi/Sb and Te1 is larger than that between Te1 and Te1, the bonding between two adjacent quintuple‐layers might be strengthened when more Bi/Sb atoms occupy the Te sites, which could accelerate phonon transfer and neutralize the *κ*_l_ reduction caused by twins’ formation. As a result, the *κ*_l_ nearly maintains unchanged as *δ* ≥ −0.02. With *δ* further decreasing, the density of twins is saturated, but the number of Bi(Sb)_Te_ increases continuously, thus leading to a *κ*_l_ increase at *δ* = 0.03. Nevertheless, benefitting from the significantly suppressed bipolar effect, the *κ*_b_ should be accordingly reduced in the Bi_0.4_Sb_1.6_Te_3+_ *_*δ*_* sample with *δ* < 0, thus reducing the *κ*_b_ + *κ*_l_, as well as the *κ*, at higher temperatures. Finally, the increased *κ*_tot_ and reduced *S* deteriorate the *zT* value of Bi_0.4_Sb_1.6_Te_3_ around 30–125 °C, but the optimum serving temperature of these samples is broadened, leading to an enhancement of average *zT* value between 30 and 250 °C, from 0.86 for Bi_0.4_Sb_1.6_Te_3_ to 1.07 for Bi_0.4_Sb_1.6_Te_2.97_ and 1.06 for Bi_0.4_Sb_1.6_Te_2.98_, shown as Figure 4f and Figure S6 (Supporting Information).

**Figure 5:**
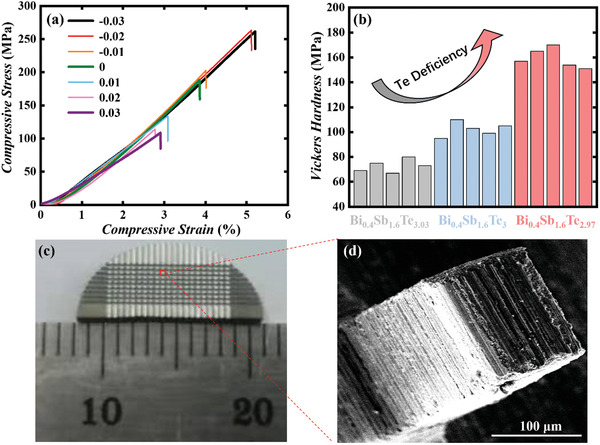
a) Compressive curves of the as‐sintered Bi_0.4_Sb_1.6_Te_3+_ *_*δ*_*, b) Vickers hardness of Bi_0.4_Sb_1.6_Te_2.97_, Bi_0.4_Sb_1.6_Te_3_, and Bi_0.4_Sb_1.6_Te_3.03_, c) Photograph of micronized bars cutting from Bi_0.4_Sb_1.6_Te_2.98_, and d) SEM images of one micronized bar.

Then, compressive testing is conducted to study the effect of nanotwins on mechanical performance. All the samples are cut into 2 × 2 × 4 mm^3^ and employed for the compressive strength measurement on MTS universal test machine at room temperature. **Figure** **5**a displays the compressing curves (strain‐stress curves) of Bi_0.4_Sb_1.6_Te_3+_ *_*δ*_* samples. Similar to compressing behavior of ceramic materials, the stress presents a linear relation with the strain until fracturing, no platform of plastic deformation can be observed. For the samples with tiny Te compensation (Bi_0.4_Sb_1.6_Te_3.01_, Bi_0.4_Sb_1.6_Te_3.02_, and Bi_0.4_Sb_1.6_Te_3.03_), the compressive strength *σ*_b_ is 140, 113, and 114 MPa, respectively, which is comparable with the value reported in nanograin (Bi, Sb)_2_Te_3_ alloys.^[^ ^34^ ^]^ As decreasing the *δ* below −0.01, the *σ*_b_ is sharply increased up to over 250 MPa, more than double higher than the *σ*_b_ of Bi_0.4_Sb_1.6_Te_3.03_. The enhanced *σ*_b_ should be ascribed to the dense twins observed in the TEM micrographs. First, the occurrence of twins is equal to further reducing the grain size, leading to increased obstructions for the dislocation slippage, i.e., the dislocations accumulate and multiply at the twin boundaries, corresponding with the Hall‐Petch relation.^[^ ^61^ ^]^ Moreover, for our samples, some interstitials located at the gaps of the twin boundaries act as pins to make the slide along the twin boundary harder, which also improves the mechanical strength. However, as *δ* ≤ −0.02, the *σ*_b_ nearly maintains unchanged due to the following reasons. Reducing Te‐rich phases by decreasing Te content is the main reason for the twin density increasing. Hence, once the Te‐rich phases completely disappear, the twin density stop to increase with the *δ* further reducing, leading to that the *σ*_b_ reaches the maximum value and no longer changes. Meanwhile, unlike the incoherent grain boundaries that only provide barriers to the dislocation transmission from one grain to the next and reduce the ductility, the coherent twin boundaries could absorb a high density of dislocations, which is much more favorable for the ability to accommodate plastic deformation.^[^ ^8^ ^]^ Hence, the compressive strain is also increased up to 5.2% in Bi_0.4_Sb_1.6_Te_2.97_ alloy, much higher than the 2.5–2.8% for the Te compensated samples. The compressive testing is repeated five times for Bi_0.4_Sb_1.6_Te_3_ and Bi_0.4_Sb_1.6_Te_2.97_ to guarantee data reliability. As shown in Figure S7 (Supporting Information), the testing results present satisfactory repeatability, the *σ*_b_ of Bi_0.4_Sb_1.6_Te_3_ and Bi_0.4_Sb_1.6_Te_2.97_ respectively varies within 160–195 MPa and 234–263 MPa. The Vickers hardness measurement is also conducted on Vickers’ microhardness tester (HVS‐1000) with a load of 0.05 kg and a loading time of 15 s for the sample of Bi_0.4_Sb_1.6_Te_2.97_, Bi_0.4_Sb_1.6_Te_3,_ and Bi_0.4_Sb_1.6_Te_3.03_, each sample is measured by five times. As shown in Figure 5b, the Bi_0.4_Sb_1.6_Te_2.97_ exhibits the highest hardness of 156 MPa (average value). Meanwhile, synthetically considering the mechanical and thermoelectric performance, we choose the sample of Bi_0.4_Sb_1.6_Te_2.98_ to examine its machinability. Only by the simple diamond wire cutting, the sample can be cut into very small bars with a cross‐section of 100 × 100 µm, shown in Figures 5c,d. It should be noted that drastic vibration exists in the cutting process and restricts the further improvement of cutting quality. We believe much finer cutting can be realized by using a more precise device. In one word, our Te‐deficiency sample possesses superhigh mechanical strength while maintaining decent thermoelectric performance, thus very attractive for the fabrication of a miniature device.

## 3 Conclusion

In this work, the twin formation mechanism is systematically investigated in a series of Bi_0.4_Sb_1.6_Te_3+_ *_*δ*_* with different Te content. Through abundant microstructure characterization, we confirm that the twins belong to annealing twins formed in the sintering process rather than the deformation twins generated by the heavy deformation (reported in previous works). Moreover, despite possessing intrinsic low stacking faults energy, heavy deformation, and low Te content, respectively providing the driving force for recrystallization in the sintering process and reducing the number of Te‐rich phases that impede the twins’ formation, are also essential to obtain dense nanotwins in Bi_0.4_Sb_1.6_Te_3_ alloys. Based on the clarified formation mechanism mentioned above, dense twins are constructed in Te‐deficient Bi_0.4_Sb_1.6_Te_3_ alloys to produce ultra‐high mechanical strength and excellent machinability. Taken together with the optimized thermoelectric properties, this sample shows great potential in practical application, especially applied for the miniature device.

## 4 Experimental Section

### Sample Preparation

According to the nominal composition of Bi_0.4_Sb_1.6_Te_3+_ *_*δ*_* (*δ* = −0.03, −0.02, −0.01, 0, 0.01, 0.02, 0.03) are fabricated, raw materials, (bismuth (Bi, 99.99%; Alfa Aesar), antimony (Sb, 99.99%; Alfa Aesar), and tellurium (Te, 99.99%; Alfa Aesar)), were weighed and sealed into quartz tubes with the vacuum condition of 10^−5^ torr. Then, these tubes were heated up to 800 °C followed by furnace cooling after 10 h dwelling. 2 h of high‐energy ball milling on SPEX 8000M was used to crush the ingots into fine powders which were immediately condensed via spark plasma sintering (SPS) in an *Φ*12.7 mm graphite die at 400 °C with an axial pressure of 80 MPa for 5 min.

### Sample Characterization

X‐ray diffraction (XRD) with Cu *K*_*α*_ (*λ* = 1.5406 Å) radiation was employed to detect the crystal structure. Specimens were prepared by conventional methods, the procedures were performed including the cutting, grinding, dimpling, polishing and Ar‐ion milling in a liquid nitrogen environment. Scanning transmission electron microscopy (TEM, and STEM) and energy‐dispersive X‐ray spectroscopy (EDS) investigation, which were conducted with JEM‐ARM300F2 and JEM‐ARM200F NEOARM in Instrumental Analysis Center of Xi'an Jiaotong University. The electrical properties (composing of the electrical conductivity and Seebeck coefficient) measurement were conducted on the ZEM‐3 setup (Ulvac‐Riko, Inc. Japan) using the four‐point dc current‐switching method and the static temperature difference method. Thermal conductivity was calculated according to the relationship *κ = Dρ*C_p_, in which *D* was the thermal diffusivity measured by a laser flash method with a commercial system (LFA 457, Netzsch), *ρ* was the density obtained through Archimedes method, *C*_p_ was the heat capacity assumed to the Dulong‐Petit limit and to be temperature independent. The measurement directions of electrical and thermal properties were all perpendicular to the sintering pressure. The Hall coefficients (*R*_H_) measurement was conducted on the van der Pauw technique under a reversible magnetic field of 1.5 T. The Hall carrier density (*n*) and Hall carrier mobility (*μ*) were calculated from the relationship *n = 1/*(*eR*_H_), and *μ* = *σR*_H_, respectively. The compressive strength was conducted on the MTS universal test machine at room temperature with prismatic bars of 2 × 2 × 4 mm^3^ at a compressing rate of 0.005mm/min. Stacking faults energy was calculated by Density functional theory (DFT) calculation, the details were presented in Support Information IV.

## Conflict of Interest

The authors declare no conflict of interest.

## Supporting information

Supporting Information

## Data Availability

The data that support the findings of this study are available from the corresponding author upon reasonable request.
