# Supplementary material for: Nanotwins Strengthening High Thermoelectric Performance Bismuth Antimony Telluride Alloys
Source: Adv Sci (Weinh). 2022 Mar 18;9(14):2200432. doi: 10.1002/advs.202200432 (PMC9108614; doi:10.1002/advs.202200432)
Supplement: Supplementary file 1 — Supporting Information [file ADVS-9-2200432-s001.pdf]

Supporting Information

Nanotwins Strengthening High Thermoelectric Performance Bismuth Antimony Telluride Alloys

Haixu Qin[1,#], Wanbo Qu[2,#], Yang Zhang[3], Yongsheng Zhang[4], Zihang, Liu[1], Qian Zhang[5], Haijun Wu[2*], Wei Cai[1], Jiehe Sui[1*]*

I. X-ray diffraction (XRD) patterns of as-sintered $Bi_{0.4}Sb_{1.6}Te_{3+\delta}$

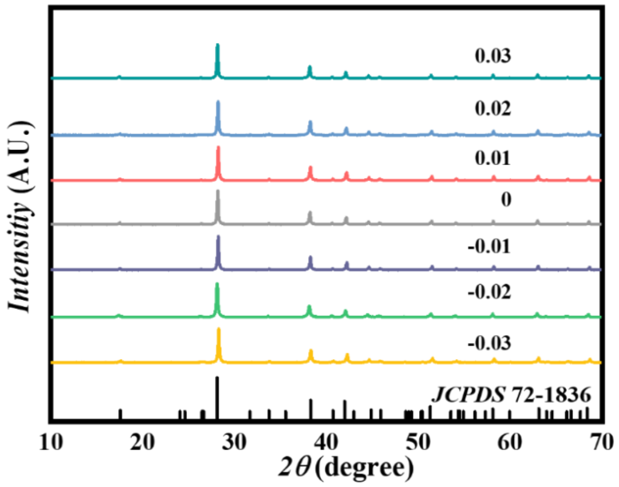

Figure S1. X-ray diffraction patterns of the as-sintered $Bi_{0.4}Sb_{1.6}Te_{3+\delta}$.

II. Microstructure of $Bi_{0.4}Sb_{1.6}Te_{3+\delta}$

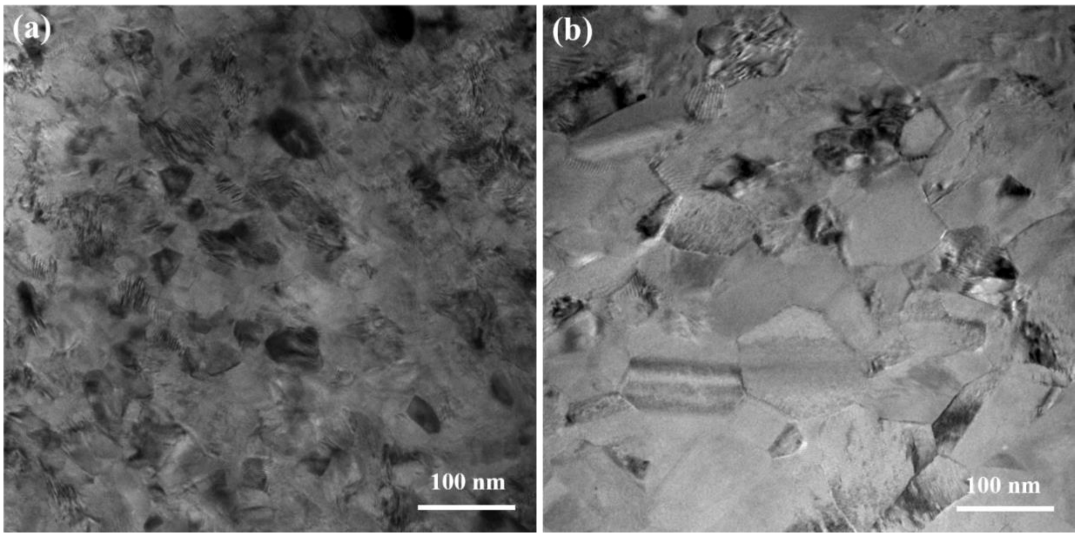

Figure S2. Low-magnification TEM images of ball-milled $Bi_{0.4}Sb_{1.6}Te_{2.97}$ sintered at 473 K. (a) and (b) are the images obtained from two different regions in the same sample.

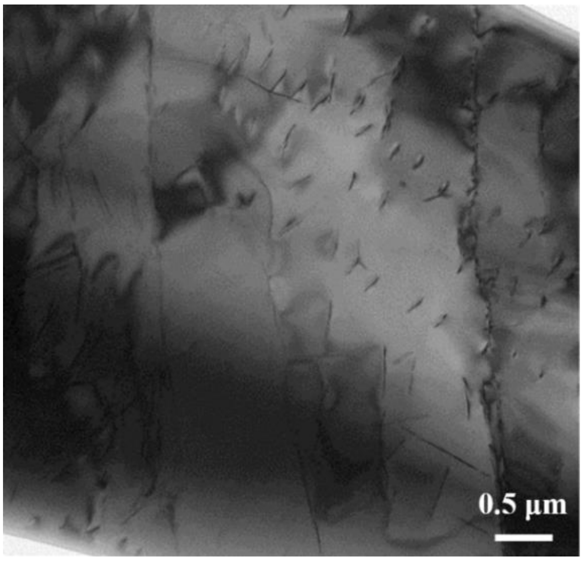

Figure S3. TEM micrographs of HM-$Bi_{0.4}Sb_{1.6}Te_3$ alloy prepared by melting + hand milling + sintering

III. Density functional theory (DFT) calculations of stacking faults

We performed the Quantum-Espresso (QE) code[1,2] with the ultrasoft pseudopotentials considering the nonlinear core corrections. The exchange-correlation functional is from the generalized gradient approximation (GGA) proposed by Perdew, Burke, and Ernzerhof (PBE).[3] The kinetic energy cutoff for wavefunctions is 50 Ry. The Brillouin zones are sampled by the Monkhorst-Pack k-mesh[4] of (8×8×1). Atomic positions and unit cell parameters are completely relaxed until all forces and components of the stress tensor are below 0.01 eV/Å and 0.2 kbar, respectively. Based on the experimental observations, we build the stacking fault Sb_2Te_3 geometry with twin boundaries (**Figure S4**). Since partial Bi (0.4) atoms are randomly occupied at the Sb sites ($Bi_{0.4}Sb_{1.6}Te_3$), we further carried out the virtual crystal approximation (VCA)[5] to simulate such random structures (or solid solution structures). The formtation energy of the stacking fault (E_f) is defined as, E_f = [E(stacking) - E(perfect)]/2A, where E(stacking) and E(perfect) are the total energies of the Sb_2Te_3 based compound with and without (the perfect crystal geometry) the stacking fault, respectively. A is the twin boundary area and the factor 1/2 is used because the cell contains two TBs. It turns out that the stacking fault energy in $Bi_{0.4}Sb_{1.6}Te_3$ is 48.9 mJ/m^2. Additionally, decreasing the Te content introduces the Te vacancy. It should be noted that Te vacancy is unstable due to the high formation energy and inclined to be occupied by the Bi or Sb atoms.[6] Hence, we investigated the stacking fault energy change with the Sb/Bi randomly substituted at the Te site as well. Once partial Bi (0.3) and Sb (0.3) is further substituted at the Te sites [$(Bi_{0.4}Sb_{1.6})(Bi_{0.3}Te_{2.7})$ and $(Bi_{0.4}Sb_{1.6})(Sb_{0.3}Te_{2.7})$], their corresponding stacking fault energies are increasing and decreasing to 57.7 mJ/m^{2}and 38.5 mJ/m^2, respectively.

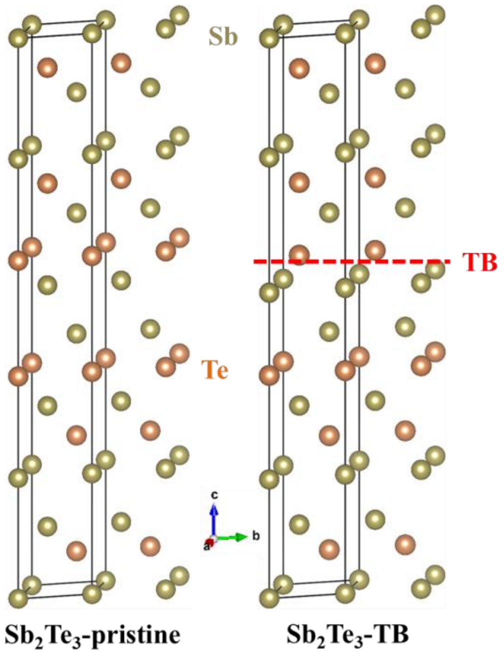

Figure. S4 Crystal structures of the Sb_2Te_3 compound without (the left panel) and with twin boundaries (the right panel). For $Bi_{0.4}Sb_{1.6}Te_3$, partial of Bi atoms (0.4) are occupied at the Sb sites. For $(Bi_{0.4}Sb_{1.6})(Bi_{0.3}Te_{2.7})$ and $(Bi_{0.4}Sb_{1.6})(Sb_{0.3}Te_{2.7})$, additional partial Bi (0.3) and Sb (0.3) atoms are occupied at the Te sites, respectively. The green and orange spheres represent the Sb and Te atoms, respectively. The red dashed line resprenents the position of the twin boundary (TB).

IV. Pisarenko plots of $Bi_{0.4}Sb_{1.6}Te_{3+\delta}$ between 30 and 250 ℃

The effective mass exhibits an upward tendency with the carrier concentration increasing. For the sample with similar carrier concentration, such as $Bi_{0.4}Sb_{1.6}Te_{2.99}$ and $Bi_{0.4}Sb_{1.6}Te_{3.01/3.02/3.03}$, there are no significant difference in the effective mass, regardless of the twin density in these samples. Hence, the increased effective should be ascribed to the convergence of multi-valley bands caused by the enlarged carrier concentration. The increased n pushes the E_F deep into the valence band, a large population of holes will form in the secondary valence to enhance the DOS near the Fermi level and then contributes to the m^* enhancement.[7]

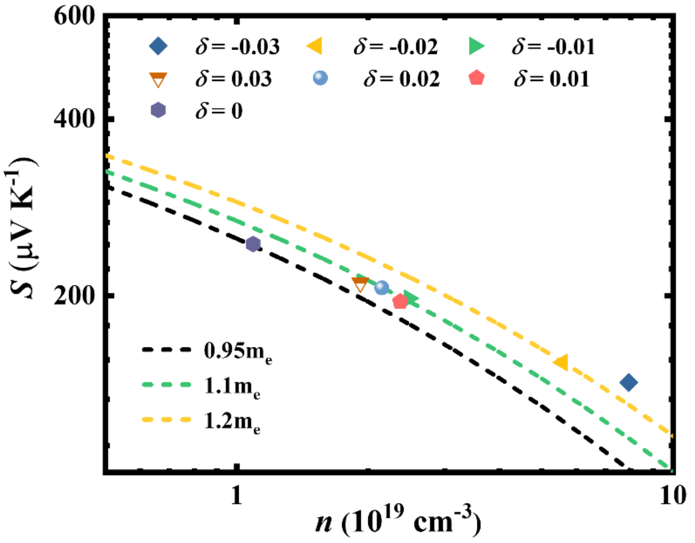

Figure S5. The Pisarenko plots of $Bi_{0.4}Sb_{1.6}Te_{3+\delta}$ (δ = -0.03, -0.02, -0.01, 0, 0.01, 0.02, 0.03)

V. Average zT value of $Bi_{0.4}Sb_{1.6}Te_{3+\delta}$ between 30 and 250 ℃

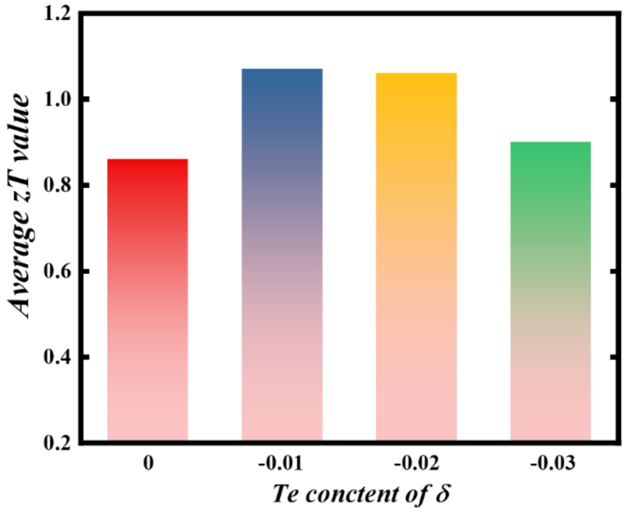

Figure S6. Average zT value of $Bi_{0.4}Sb_{1.6}Te_{3+\delta}$ between 30 and 250 ℃

VI. Hall measurement results of the samples in this work

Table S1. Carrier concentration and mobility of our samples at room temperature

Sample	n ($10^{19}cm^{-3}$)	μ ($cm^2V^{-1}s^{-1}$)
$Bi_{0.4}Sb_{1.6}Te_3$	1.09	374.9
$Bi_{0.4}Sb_{1.6}Te_{2.99}$	2.48	222.0
$Bi_{0.4}Sb_{1.6}Te_{2.98}$	5.58	139.4
$Bi_{0.4}Sb_{1.6}Te_{2.97}$	7.91	107.8

VII. Repeated compressive testing for $Bi_{0.4}Sb_{1.6}Te_3$ and $Bi_{0.4}Sb_{1.6}Te_{2.97}$

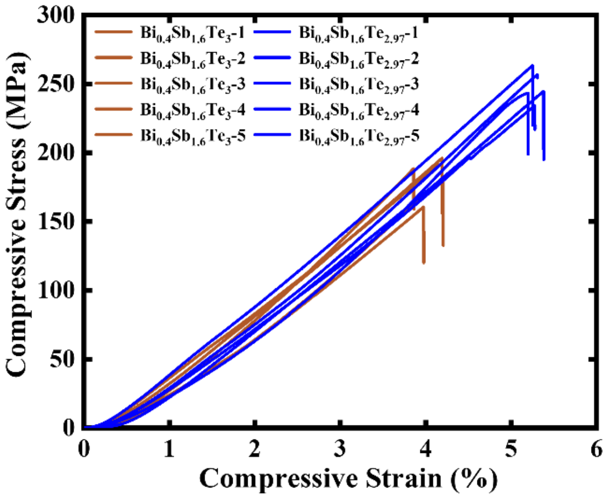

Figure S7. Compressive curves of $Bi_{0.4}Sb_{1.6}Te_3$ and $Bi_{0.4}Sb_{1.6}Te_{2.97}$ repeatedly measured by five times.

Reference

1 Giannozzi, P. *et al.* Quantum Espresso: a modular and open-source software project for quantum simulations of materials. *J. Phys. Condens. Matter.* **21**, 395502 (2009).

2 Giannozzi, P. *et al.* Advanced capabilities for materials modelling with Quantum Espresso. *J. Phys. Condens. Matter.* **29**, 465901 (2017).

3 Perdew, J. P. *et al.* Generalized Gradient Approximation Made Simple. *Phys. Rev. Lett.* **77**, 3865-3868 (1996).

4 Monkhorst, H. J. & Pack, J. D. Special points for Brillouin-zone integrations. *Physical Review B* **13**, 5188-5192 (1976).

5 Nordheim, L. Zur Elektronentheorie der Metalle. I. *Annalen der Physik* **401**, 607-640 (1931).

6 Hashibon, A. & Elsässer, C. First-principles density functional theory study of native point defects in Bi_2Te_3. *Phys. Rev. B* **84**, 144117 (2011).

7 H. Qin *et al.*, Critical role of tellurium self-compensation in enhancing the thermoelectric performance of p-Type $Bi_{0.4}Sb_{1.6}Te_3$ alloy. *Chemical Engineering Journal* **425**, 130670 (2021).
